# Bisphosphonate-Induced Acute Orbital Inflammation in a Patient With Underlying Thyroid Ophthalmopathy

**DOI:** 10.7759/cureus.12024

**Published:** 2020-12-11

**Authors:** James Yu, Jian Guan

**Affiliations:** 1 Internal Medicine, AdventHealth Orlando, Orlando, USA

**Keywords:** orbital inflammation, alendronate, osteoporosis, graves' orbitopathy

## Abstract

Bisphosphonates are widely used for various conditions, including osteoporosis, hypercalcemia of malignancy, osteolytic bone metastasis, and Paget's disease. Bisphosphonate-induced orbital inflammation is a rare side effect of amino-bisphosphonates. There has been less focus on the risk of developing amino-bisphosphonate-induced orbitopathy in people who have underlying ophthalmopathy. Herein, we present a case of alendronate-induced acute orbital inflammation in a patient with underlying Graves’ ophthalmopathy. Soon after administration of intravenous (IV) dexamethasone with topical prednisolone, the inflammation rapidly resolved. To our knowledge, this is the first case of bisphosphonate-induced orbital inflammation with underlying orbitopathy. This case demonstrates that systemic corticosteroids can be an effective treatment in orbital inflammation in similar cases. There is a possible interaction of T-cell and cytokine involvement mechanisms between Graves’ orbitopathy and bisphosphonate-induced orbital inflammation. This case also shows that bisphosphonate-induced acute orbital inflammation is rare but should be part of a physician's differential diagnosis, and more precautions are necessary for patients with underlying orbitopathy who are taking bisphosphonates.

## Introduction

Bisphosphonates are the most commonly used medication to treat osteoporosis [1]. They are also widely used for hypercalcemia of malignancy, osteolytic bone metastasis, and Paget’s disease. Amino-bisphosphonates, the newer generation bisphosphonates, contain a nitrogen group that has been reported to activate gamma delta (γδ) T-cells and lead to phase reaction [2]. It has been suggested that this mechanism in amino-bisphosphonates can cause ocular inflammation, although rarely [3]. In recent years, there have been multiple reports of inflammatory ocular side effects from amino- bisphosphonates. However, there has been less focus on the risk of developing amino-bisphosphonate-induced orbitopathy in people who have underlying ophthalmopathy. Herein, we report a case of orbital inflammation after alendronate intake in a patient who had well-controlled underlying thyroid ophthalmopathy.

## Case presentation

A 62-year-old woman presented to the emergency room (ER) complaining of a nine-day history of acute right eye pain and swelling that started within 24 hours of taking alendronate. She had a history of Graves’ disease status post-radiation ablation and was on levothyroxine. She also reported chronic bilateral proptosis with an unsure onset and had not been on any treatment for the underlying proptosis, including steroids, before admission.

**Figure 1:**
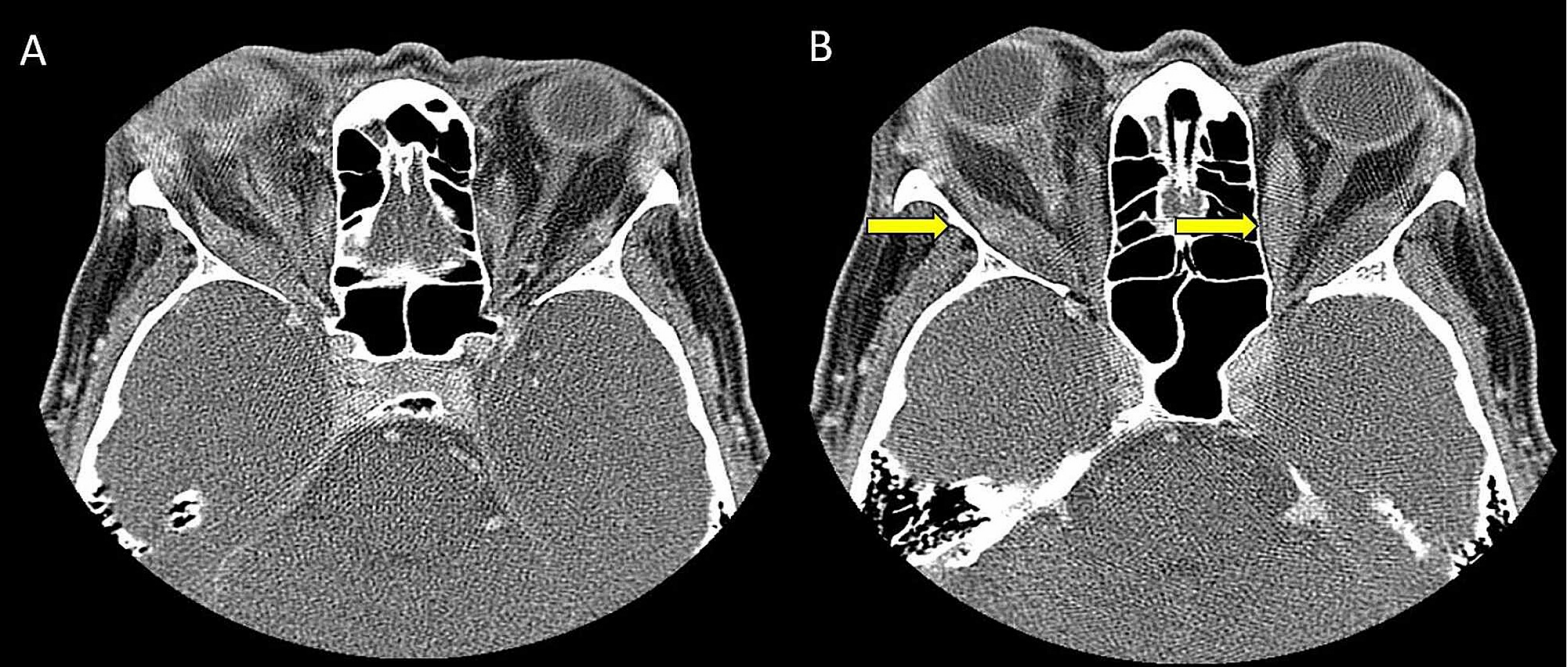
Right orbital inflammation with bilateral underlying thyroid orbitopathy A) Right orbital sclero-uveitis and pre and post-septal cellulitis with the involvement of the optic nerve sheath; B) diffuse enlargement of the extraocular muscles bilaterally, worst in the right lateral rectus muscle belly, which is suspicious for myositis. The left extraocular muscle enlargement and proptosis are consistent with underlying thyroid orbitopathy.

Ten days before coming to the emergency department (ED), she took the first dose of alendronate for her recently diagnosed osteoporosis. The next day the patient noticed immediate right eye pain and swelling. She was seen by an ophthalmologist on that day and was prescribed moxifloxacin and prednisolone acetate eye drops. However, after taking a second dose of alendronate two days earlier, she had increased right eye pain and swelling without any symptoms in the left eye which prompted her to visit the ED. Ophthalmology was consulted and a physical examination of the right eye revealed a 3+ periocular edema with erythema, consistent with conjunctivitis. Visual acuity was 20/200 in the right and 20/50 in the left which was her baseline. Computed tomography (CT) of the orbit with contrast revealed right orbital scleritis, pre and post-septal cellulitis with the involvement of the optic nerve sheath concerning the right orbit vitritis, and myositis (Figure 1). Physical examination of the left eye showed proptosis without signs of inflammation. The CT revealed extraocular muscle enlargement without acute infection or inflammation, which was consistent with underlying Graves’ orbitopathy, as evaluated by a radiologist and an ophthalmologist (Figure 1). Thyroid-stimulating immunoglobulin was increased (> 500) without a known baseline. Free T4 and thyroid-stimulating hormone (TSH) were within the normal range.

The patient was admitted to the hospital and we started IV dexamethasone, topical prednisolone, cyclopentolate, as well as empirical antibiotics of intravenous vancomycin and piperacillin/tazobactam. After two days, right eye pain and inflammation were significantly improved with continued intact visual acuity. We stopped empiric antibiotics due to low suspicion of infection and the patient was discharged with oral indomethacin and topical cyclopentolate.

## Discussion

This is the first case report of a patient with underlying orbitopathy who developed a superacute onset of orbital inflammation within 24 hours of taking a dose of oral bisphosphonate.

A recent literature review analyzing 68 patients of bisphosphonate-induced orbital inflammation demonstrated that intravenous amino-bisphosphonates cause more orbital/ocular complications, 82% (60/68), than oral amino-bisphosphonates, 12% (6/68), in their pool [4]. Considering that oral amino-bisphosphonates are more often compared to intravenous amino-bisphosphonates, ocular inflammation is even less common among patients with oral bisphosphonates than IV bisphosphonates. Among seven cases of alendronate-induced orbital inflammation in the literature review, the general onset of symptoms (mean) was 19.6 days (range: 10 to 28 days) with a final diagnosis of scleritis in three, myositis in one, and uveitis in three and no involvement of the optic sheath or septal cellulitis [4]. In our case, the symptoms developed within 24 hours. The orbital inflammation was more severe with multiple conditions: scleritis, conjunctivitis, pre and post-septal cellulitis with the involvement of the optic nerve sheath, vitritis, and myositis. Those findings suggest that underlying Graves' ophthalmopathy is a risk factor of bisphosphonate-induced ocular inflammation. They also suggest that orbital inflammation could be more rapidly and severely involved in patients with underlying Graves’ orbitopathy. Our case also demonstrated that even in bisphosphonate-induced orbital inflammation with underlying Graves’ orbitopathy, glucocorticoids are a considerable option.

Amino-bisphosphonates are known to activate a subset of T-cells known as gamma delta T-cells [2]. They initiate an acute inflammatory response from the activation of T-cells and cytokine release and have been hypothesized as the mechanism for bisphosphonate-induced orbital inflammation [5]. In Graves’ orbitopathy, TSH receptor antibodies and activated T-cells also play important roles [6]. The autoimmune process by TSH receptor antibodies induce retro-ocular adipogenesis, an inflammatory infiltrate, and glycosaminoglycan (GAG) accumulation [7]. Increased activity of T-cells also plays a role in the development of orbitopathy by Th1 lymphocyte-producing cytokines-induced fibroblast proliferation and GAG production, as well as by Th2 lymphocyte remodeling and fibrosis of periorbital tissues in the late phase [7]. Considering how both mechanisms involve T-cell and cytokine release, it is possible that underlying Graves’ orbitopathy can promote bisphosphonate-induced orbital inflammation. It is also notable that anti-inflammatory drugs, especially glucocorticoids, have been suggested as proper treatment for bisphosphonate-induced orbital inflammation, which is also a typical treatment for Graves’ orbitopathy.

## Conclusions

Our case report suggests that orbital inflammation could be more rapidly and severely involved in patients with underlying Graves’ orbitopathy. There is a possible interaction of T-cell and cytokine involvement mechanism between Graves’ orbitopathy and bisphosphonate-induced orbital inflammation. Although it is rare, this case reminds us that bisphosphonate-induced acute orbital inflammation should be part of a physician’s differential diagnosis, especially for patients with underlying orbitopathy. Further research would be necessary regarding the precaution of bisphosphonate-induced orbital inflammation in patients with underlying Graves’ orbitopathy.
